# New Insights on Metabolic and Genetic Basis of Migraine: Novel Impact on Management and Therapeutical Approach

**DOI:** 10.3390/ijms23063018

**Published:** 2022-03-11

**Authors:** Irene Simonetta, Renata Riolo, Federica Todaro, Antonino Tuttolomondo

**Affiliations:** 1 Internal Medicine and Stroke Care Ward, Department of Promoting Health, Maternal-Infant Excellence and Internal and Specialized Medicine (ProMISE) G. D’Alessandro, University of Palermo, Piazza delle Cliniche n.2, 90127 Palermo, Italy; irene.simonetta@live.it (I.S.); renatariolo.rr@gmail.com (R.R.); federicatodaro21@gmail.com (F.T.); 2 Molecular and Clinical Medicine PhD Programme, University of Palermo, P.zza delle Cliniche n.2, 90127 Palermo, Italy

**Keywords:** migraine, genetics, neurobiology, cortical spreading depression

## Abstract

Migraine is a hereditary disease, usually one-sided, sometimes bilateral. It is characterized by moderate to severe pain, which worsens with physical activity and may be associated with nausea and vomiting, may be accompanied by photophobia and phonophobia. The disorder can occur at any time of the day and can last from 4 to 72 h, with and without aura. The pathogenic mechanism is unclear, but extensive preclinical and clinical studies are ongoing. According to electrophysiology and imaging studies, many brain areas are involved, such as cerebral cortex, thalamus, hypothalamus, and brainstem. The activation of the trigeminovascular system has a key role in the headache phase. There also appears to be a genetic basis behind the development of migraine. Numerous alterations have been identified, and in addition to the genetic cause, there is also a close association with the surrounding environment, as if on the one hand, the genetic alterations may be responsible for the onset of migraine, on the other, the environmental factors seem to be more strongly associated with exacerbations. This review is an analysis of neurophysiological mechanisms, neuropeptide activity, and genetic alterations that play a fundamental role in choosing the best therapeutic strategy. To date, the goal is to create a therapy that is as personalized as possible, and for this reason, steps forward have been made in the pharmacological field in order to identify new therapeutic strategies for both acute treatment and prophylaxis.

## 1. Introduction

Migraine is a central nervous system disorder that ranks in the top 5 medical disabling disorders contributing to years lived with disability; it affects roughly 1 billion subjects worldwide [1].

Migraine is a neurological condition characterized by headache, often unilateral, of moderate to severe intensity and often associated with nausea, vomiting, photophobia, and phonophobia [2].

Migraine is defined as episodic when attacks of headache manifest less than 15 days per month, chronic if the attacks are occurring for 15 or more days per month for more than 3 months with at least 8 days manifesting typical migraine characteristics. Moreover, migraine is classified as with or without aura [3,4].

Aura is a set of reversible neurological disturbances occurring before or during a migraine episode, including sensory, visual, and speech disorders that are usually aphasic and difficult to characterize. Visual aura constitutes the most common type of aura with multifaceted features. Clinical studies of visible aura symptoms have reported many heterogeneous, sometimes complex, visual disturbances: the perceived optical phenomena that are frequently described include zigzag lines, crescent shapes, and flickering lights [5].

Sensory disturbances may occur as paresthesias, as needle sensation that starts from a point in the hand and slowly migrates to the arm; it may also involve the face, lips, and tongue: these sensations may be accompanied by numbness. Besides aura, a prodromal phase of a migraine attack may also comprise several symptoms, such as fatigue, pallor, and yawning obfuscating vision. Finally, after resolution of headache, a postdrome degree may manifest with hyporeactivity, hyper-reactivity, depression, uncontrollable desire for a specific food, and depression.

According to a review by Burch et al., 1 in 6 people suffer from migraines in the United States: the affected subjects are young or middle-aged, and the prevalence of migraine is significant in the age range of 18 to 44 years. Its prevalence declines with ageing. In fact, in this sample prevalence was 15.9% in people aged 45 to 64 years and 7.3% in the age range of 65–74, followed by 5.1% [6].

In this work, the authors reported differences concerning the prevalence of migraines when they examined other factors, such as gender, work status, ethnicity, income, insurance: the highest prevalence in women, in those people living below an annual household income of $35,000, in those people who were unemployed but with a previous job (21.4%) compared with those who were unemployed and had never worked (16.6%), and in people with a full-time job (13.3%) compared with the subject having a part-time job (15.6%). As concerns ethnicity, native Americans were the most affected as compared with black, white, and Hispanic individuals [7,8].

In 2013, migraine was globally classified as the sixth cause of disability [3].

The risk factors of migraine may be nonmodifiable and modifiable. The first risk factor includes genetics, gender, and age: a probability of 40% of manifesting migraine has been reported in those with a parent with migraine and 75% in subjects with both parents affected by migraine. Although, as noted above, adult women experience migraine three times more likely than men, these data are not applied in adolescence, in which migraine is more common in boys.

Modifiable factors involved in the risk of progression from episodic to chronic migraine include obesity, stressful events, and medication overuse; additionally, migraine triggers exist and are patient specific. They contain caffeine and food additives, and missed and delayed meals may be reported as a possible catalyst. In the light of these last considerations, a significant role of health-care professionals is also educating patients on methods, protective behavior such as physical exercise, use of preventive medications, management of stressful life events, and specific diets. To establish whether an item may constitute a trigger, a patient should avoid it for a minimum of 4 weeks and then start with a slow reintroduction of the suspected trigger [9,10,11].

Metabolic alterations represent another face of migraine pathophysiology. It is clinically known that several metabolic factors may trigger migraine, such as fasting and lack of sleep. The underlying molecular mechanisms include increased oxidative stress, alterations of mitochondrial metabolism, impaired glucose metabolism, and reduction of some metabolic substrates in the cerebral cortex [12,13,14,15].

All these abnormalities at least partially seem to be associated with a genetic basis. For example, genes that code for metabolic enzymes and are expressed in both mitochondrial and nuclear DNA are more related to the pathogenesis of migraine [16,17].

Metabolic treatments, thus, may act on the biochemical alterations that characterize the nervous system of migraineurs. Responsible alterations of the migraine attack have been found at the beginning of the pathogenetic cascade.

In recent years, major progress has been made in the knowledge of the pathophysiologic basis of migraine, allowing the development of targeted therapeutic strategies.

## 2. Updates on Migraine Pathophysiology

The pathophysiology of migraine is a wide and complex territory still not completely clear. The anatomy and physiology of the nervous structures involved in the genesis of pain and the pathways that regulate it should be analyzed to understand the pathophysiological mechanisms underlying migraine. According to electrophysiology and imaging studies, many brain areas are involved, such as cerebral cortex, thalamus, hypothalamus, and brainstem [18,19,20,21,22].

Several studies have formulated various hypotheses, and there is still significant debate regarding neural- versus vascular-based migraine models; however, the exact mechanisms of migraine are still undetermined. A well-described mechanism involved in migraine is the activation of the trigeminovascular system [23,24,25], which has a key role in the headache phase. Concerning the migraine aura phase, which occurs in approximately one-third of individuals with migraine, recent evidence supports the hypothesis that cortical spreading depression (CSD) is the underlying physiologic process.

### 2.1. Cortical Spreading Depression

Cortical spreading depression (CSD) is a slowly propagating wave of depolarization in neuronal and glial cell membranes. It was described for the first time in 1944 by Aristides Leão. He described it as a reduction in the spontaneous electrical activity of the cortex in a rabbit model of experimental epilepsy [26].

The slowly depolarizing wave is followed by inhibition of cortical activity for up to 30 min, and depression is also associated with a wave of hyperemia, followed by a prolonged phase of cortical oligemia [27].

CSD causes various modifications in the local microenvironment, such as a dysregulation of adenosine triphosphate, glutamate, potassium, nitric oxide, and CGRP dynamics. According to recent evidence, repeated depolarization and repolarization of hyperexcitable neurons in the cerebral cortex is responsible for the initial accumulation of extracellular K^+^. This K^+^ extracellular accumulation further depolarizes the cells that initially release it. It is associated with major disruption of cell membrane ionic gradients, the influx of sodium (Na^+^) and calcium (Ca^2+^), and glutamate release [28,29,30,31]. The propagation of CSD is still unclear, and various authors have formulated several hypotheses. Initially, K^+^ extracellular accumulation or anomalous glutamate diffusion was thought to lead to the propagation of CSD. Later, other authors indicated the gap junctions between neurons and glial cells as responsible for CSD propagation [32].

This depolarization wave causes changes in cerebral blood flow and oxygenation; in fact, CSD causes vasodilatation and a consequent phenomenon called spreading hyperemia, a term that indicates an increase in regional cerebral blood flow. Spreading hyperemia has a duration of 1–2 min, and it is followed by spreading oligemia. This is a prolonged phase of hypoperfusion with a duration of 1–2 h [33]. In association with the increased metabolic needs due to CDS, these changes lead to dysregulation of normal mechanisms of cerebral homeostasis and to a mismatch of supply and demand.

According to some authors, CSD seems to be correlated to the activations of meningeal nociceptors. In fact, some evidence from animal studies supports the idea that molecules released during CSD, such as glutamate, ATP, K1, CGRP, hydrogen ions, and nitrous oxide, could activate meningeal nociceptors [34]. Even though the action of CSD on the trigeminovascular system is still debated, some studies on the visual cortex in mice showed that focal stimulation induced CSD and consequently caused the release of proinflammatory molecules from neurons and astrocytes by opening neuronal pannexin 1 megachannels, resulting in the activation of meningeal nociceptors. Furthermore, suppression of this pannexin-1-dependent cascade abolished CSD-induced trigeminovascular activation, dural mast cell degranulation, and headache. This suggests the possibility that CSD-induced neuronal meg-channel opening could promote trigeminal-mediated nociception in migraine via inflammatory cascades [35]. In addition, it has been shown that CSD-induced increases the activity of trigeminovascular neurons in the spinal trigeminal nucleus of anesthetized rats. This evidence supports the theory that CSD induces sequential activation of peripheral and then central trigeminovascular neurons. Preclinical evidence also suggests that CSD may directly activate or disinhibit central trigeminal sensory neurons by an intrinsic mechanism to the CNS. In these experiments, sensory blockade of the trigeminal ganglion did not stop CSD action on second-order trigeminovascular neurons in the TCC. Therefore, trigeminovascular neurons’ increased activity is induced by CSD not only by a peripheral action but also by a central mechanism, which could be underlying migraine pain. However, it is still unclear how changes in cerebral blood flow generated by CSD are associated with migraine aura and headache physiology. Therefore, a correlation between CSD and the initiation and progression of aura symptoms is described. In fact, the delay between CSD and this neuronal activation is approximately 14 min, which is similar to the time that elapses between the onset of aura and that of migraine headache [36]. This suggests that CSD could be a substrate of migraine with aura.

It has been suggested also that aura could be the result of an aberrant “brain state” that occurs in a genetically susceptible individual during a migraine attack, and that physiological changes occurring in the premonitory phase (which is developed before aura) may be the primary cause of both trigeminovascular pathway activation and cortical neuronal/glial activity [37,38].

### 2.2. Neurobiology: The Trigeminovascular System

Neurogenic inflammation is one of the factors implicated in the pathogenesis of several neurological diseases, such as migraine. 

It is hypothesized that protein extravasation occurs through the fenestrated endothelium of the vessels in the dura mater, thus contributing to a dural neurogenic inflammation that is also activated by neuropeptides released by perivascular nerve fibers [39].

In 1984, Moskowitz proposed the trigeminovascular system as the anatomical and physiological substrate of migraine pain. In this model, migraine pain depends on nociceptive transmission and activation of the trigeminovascular system. The activation of nociceptors innervating pial, arachnoid, and dural blood vessels leads to the headache phase of a migraine attack [40,41].

Nociceptive innervation of intracranial vasculature and the meninges consists of nonmyelinated (C-fibers) and thinly myelinated (Ad fibers) axons containing vasoactive peptides, such as calcitonin gene-related peptide (CGRP), substance P, neurokinin A, and pituitary adenylate cyclase-activating polypeptide [34]. The release of these vasoactive peptides is correlated to the induction of inflammatory reactions [42].

**Figure 1:**
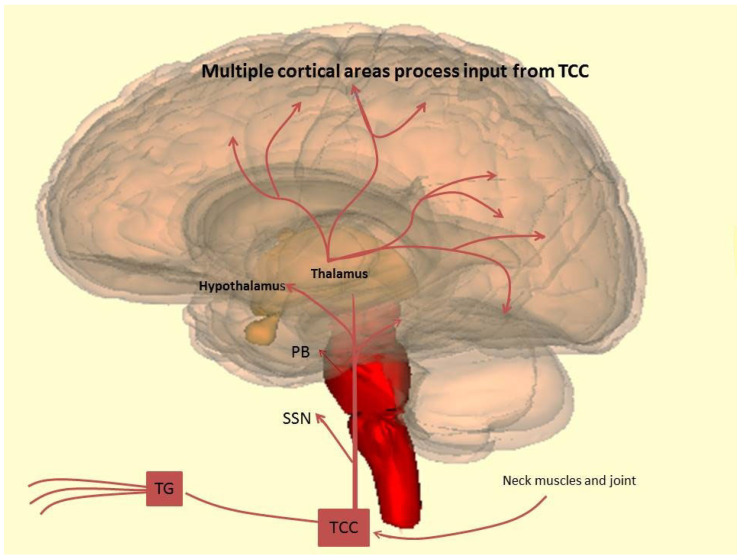
Multiple cortical areas process input from TCC.

The activation of the trigeminovascular pathway conveys nociceptive information from the meninges to the central areas of the brain, and in turn to the cortex (Figure 1).

Recently, several works have suggested a mismatched communication of the trigeminovascular system that causes sensitization and the release of CGRP; moreover, they have reported the role of some ion channels and neuropeptides and their action on the modulation of the excitability of nervous fibers (trigeminal C neurons and the Aδ neurons) [39].

The study of molecular mechanisms behind migraine may lead to the detection of novel approaches aiming at modifying the system and developing new therapies.

It has been proposed that the origin of migraine pain is a pain-transmitting fiber: Aδ neurons.

These fibers are the only ones to express CGRP receptors peripherally. This hypothesis comes from the observation of success and efficacy of new monoclonal antibodies targeting CGRP/CGRP receptors. Moreover, Burstein reported that the antimigraine effects of CGRP monoclonal antibodies are also exerted by blocking the bind between CGRP and trigeminal Aδ neurons [43,44].

After being linked by CGRP, the Gαs subunit of a CGRP receptor activates a cascade that leads to the conversion of ATP into cAMP. The higher cytosolic cAMP levels cause the activation of PKA (protein kinase A), which regulates ion channels. CGRP indirectly regulates the propagation of a nerve signal and participates in the mechanism of neural sensitization and pain transmission through this signaling pathway [45,46].

Aδ fibers become hyperexcitable for other stimuli because of the rise of cAMP concentration induced by CGRP. Moreover, the influence on the activity of the myelinated Aδ neurons at the nodes of Ranvier from CGRP has been observed. In the light of these findings, the trigeminal system appears to tune the trigeminal pain, representing an important target of novel medication (gepants and mAbs). CGRP binds its receptor expressed also on myelinated Aδ fibers localized to the nodes of Ranvier, probably causing an increment of cAMP and other components of this signaling pathway as PKA and CREB, resulting in the excitation of the neurons and likely modulating the sensitization process [47].

Nociception originates from the sensitization of first-order trigeminovascular neurons, of which cell bodies are in the trigeminal ganglion. Nociceptive transmission comes from the trigeminal ganglion to the brain stem, subsequently sensitive second-order neurons, including the trigeminal spinal nucleus. In turn, the third-order neurons in the thalamus are activated, and the nociception reaches the somatosensory cortex and other cortical areas. Almost all cranial tissues, including the intracranial and extracranial structures of the head and face, are innervated by trigeminal afferent arising in the trigeminal ganglion; occipital regions are innervated by additional cervical afferents from the upper cervical dorsal root ganglion [48]. Between the three divisions of the trigeminal nerve, the ophthalmic division is considered the most involved in migraine pain. It is responsible for the distribution of head pain around the periorbital dermatome. Part of the trigeminovascular afferents projects to the trigeminocervical complex. However, in addition to the established indirect parabrachial innervation from the trigeminocervical complex, recent studies have shown direct projections from trigeminal ganglion afferent synapse in the ipsilateral parabrachial nucleus of mice [49]. These projections could explain the presence of autonomic features and the heightened perception of painful stimuli to the face [50]. This evidence suggests that the parabrachial nucleus is innervated by two alternate pathways arising from craniofacial nociceptors and may in part explain the heightened perception of pain in the head and face. This trigemino–parabrachial–limbic pathway is responsible for processing affective-motivational aspects of pain. In the central nucleus of the amygdala, synapses have some CGRP-expressing neurons afferent from the parabrachial nucleus. Their action is to reduce appetite and modulate conditioned taste aversion [51,52]. In the sphenopalatine ganglion, there are CGRP-immunoreactive fibers and CGRP receptors. This conveys parasympathetic outflow to the head and face. According to this evidence, it seems possible that trigeminovascular afferents expressing CGRP project directly from the trigeminal ganglion to the sphenopalatine ganglion [53] (p. 5) [54,55]. The other hypothesis is that a direct connection between the trigeminocervical complex and superior salivatory nucleus pathway provides the primary parasympathetic projections to the sphenopalatine ganglion, forming a trigeminal autonomic reflex [56]. Overall, the presence of a direct ascending trigeminocervical complex to the sphenopalatine ganglion/superior salivatory nucleus projection is not still demonstrated.

Recent human studies on cluster headache, in which direct stimulation of the sphenopalatine ganglion activated the parasympathetic outflow to the face, suggest that the activation of the peripheral branch of the trigeminal autonomic reflex alone is not enough to induce head pain [57]. The trigeminocervical complex transmits sensory information to several crucial nuclei throughout the brain [58,59,60,61,62,63,64,65,66]. In particular, dural nociceptive fibers are projecting to the ventral posteromedial thalamic nuclei [40,67,68] and spinal dorsal horn neurons targeting the ventral posterolateral nuclei. Furthermore, trigeminal inputs also synapse in thalamic nuclei and in important medullary regions, such as rostral ventromedial medulla, nucleus raphe magnus, and parabrachial. In addition, they project to the brainstem (ventrolateral periaqueductal grey, nucleus of the solitary tract, and locus coeruleus) and diencephalic (lateral, anterior, and posterior hypothalamic nuclei) regions.

The thalamus plays a crucial role in multimodal sensory integration in migraine [69]. In fact, the thalamus has an important role in nociceptive, touch, visual, olfactory, and auditory sensation. Based on this evidence, the thalamus seems to be a useful target for modulating the multimodal sensory dysfunction of migraine. Studies on rats have demonstrated that retinal (light sensitive) and trigeminovascular (dural nociceptive) projections converge in the posterior thalamus of rats. It has been shown that light stimuli facilitate trigeminovascular nociceptive activation in thalamocortical relay neurons. This could be correlated with the development of photophobia [70].

Furthermore, it has been shown that tactile and thermal stimuli enhance pulvinar thalamic activity in patients with migraine suffering from extracephalic allodynia. According to these data, there is a connection between the thalamus and the presence of extracephalic allodynia during migraine attacks [71,72]. The trigeminovascular system is subject to a powerful modulation in the genesis of head pain. This modulation takes place at different levels, mostly coming from corticofugal projections to multiple brainstem regions, including the periaqueductal grey, pontine, raphe, and medullary reticular nuclei [73,74]. Although direct corticotrigeminocervical complex projections originate largely in the primary somatosensory cortex, it has been shown that visual and insular cortices have a key role in the modulation of trigeminocervical complex neuronal activity.

Other approaches for investigating the pathophysiology of migraine are represented by functional MRI studies and PET studies, particularly during a triggered or spontaneous migraine attack.

These findings have shown modifications in the hypothalamic activity likely explaining some phenomena preceding headache, such as polyuria, alterations in mood, and appetite.

Additionally, PET studies have reported a correlation between a high activity in the occipital cortex and light sensitivity, and an activated function of the brainstem has been related to nausea [75,76].

Resting-state MRI has been performed to analyze changes in the network connecting different brain areas before and during an attack. These studies have found alterations in the connectivity of the cortex, thalamus, hypothalamus, brainstem, cerebellum, amygdala that are related to abnormal functions of several overlapping circuits regulating sensory sensitivity and pain [77].

These studies, together with electrophysiological studies concerning thalamic and thalamocortical circuits, support the role of the thalamus and of the activity in thalamocortical circuits as an important player in the abnormal sensory processing characterizing a migraine attack [78].

Neuropeptides and their receptors are currently promising targets for the management of migraine.

## 3. Neuropeptides

Calcitonin gene-related peptide (CGRP) is a vasoactive neuropeptide with various cardiovascular, digestive, and sensory functions. It is a 37-amino acid neuropeptide encoded by the calcitonin gene (CALCA). CGRP and its receptor are expressed throughout the body, particularly in the central and peripheral nervous systems, gastrointestinal system, and cardiovascular system. Human CGRP exists in two isoforms: α-CGRP and β-CGRP [79,80]. α-CGRP is extensively distributed in the central (CNS) and peripheral nervous systems (PNS) [55]. A great part of the cranial vasculature is innervated by α-CGRP-containing C and Aδ sensory nerve fibers [39,55]. Beta-CGRP differs from α-CGRP by three amino acids. It is located in the enteric nerve terminals [81].

Several studies have demonstrated that CGRP is involved in migraine pathogenesis. This neuropeptide can act in both the periphery to enhance nociceptor sensitization and the CNS to enhance sensory input. It has a crucial role in pain generation, particularly in the activation of neuronal sensitization. It is a neurotransmitter, and its activity can increase synaptic transmission mediated by glutamatergic signaling.

Weiller and his group, in the mid-1990s, using high-resolution positron emission tomography, demonstrated that the blood flow in brainstem nuclei and the cerebellum is increased during spontaneous migraine attacks. They called the brainstem nuclei involved in migraine “migraine generators”, indicating the locus coeruleus (LC), the raphe nuclei, and the periaqueductal grey matter (PAG) [62].

CGRP is expressed in migraine-related structures, such as the TNC, the TRIG, the human LC, and a part of the cervical spinal cord [81,82]. The receptor activity-modifying protein (RAMP) 1 and the calcitonin receptor-like receptor (CLR), are widely expressed in the nerve fibers in the TNC [83].

Recent data suggest that the cerebellum and the CGRP have a role in the modulation of pain [43,84,85].

Several pieces of evidence hypothesize a crucial role of CGRP in migraine pathogenesis. It is also a potent vasodilator and acts in nervous terminations in meningeal blood vessels. CGRP contributes to vasodilation via two pathways: the release of CGRP leads to nitric oxide-independent vasodilation by a direct action on smooth muscle cells, but it can also stimulate endothelial production of NO. Various studies suggest that neuronal depolarization and, consequently, CSD wave lead to vasodilatation and oligemia by a central mechanism induced by the upregulation of CGRP. This evidence, in vivo and in vitro, supports the hypothesis that CSD influences CGRP activity in migraine. CGRP has a very potent endogenous vasodilatory action in the cerebral vasculature [39]. During activation of the TS, CGRP induces neurogenic inflammation in the meningeal vasculature and to mast cell degranulation through vasodilatation and plasma protein extravasation. This process leads to peripheral sensitization with clinical manifestation, such as the throbbing nature of the migraine headache and a worse headache during physical activity during migraine attack [23,86,87,88,89,90]. In the brainstem, CGRP induces central sensitization of the second-order neurons in the TNC and in the third-order neurons in the thalamus [86,91]. This process causes cephalic and extracephalic allodynia clinically [23,92]. It has also proinflammatory action. During the neuronal activation of the TRIG, CGRP is released. This stimulates the SGCs, which release proinflammatory cytokines, with modulation of the neuronal response [86,93,94,95]. According to this observation, the intraganglionic signaling process could be investigated through a new approach.

Recent evidence indicates CGRP as a key factor in the generation of pain in migraine: a study showed that high levels of CGRP can be detected in jugular venous blood during migraine attacks, and it also seems that intravenous injection of CGRP triggers migraine in patients with migraine, but not in healthy volunteers [96,97,98]. Actually, the role of CGRP as a biomarker in migraine is debated. An increased plasma level of CGRP has been observed in nitroglycerine (NTG)-induced migraine attacks. Higher levels of CGRP have also been detected in the saliva and in the serum of the cranial outflow at the cubital vein and external jugular during spontaneous migraine attacks [23,99,100,101,102]. An increase in CGRP levels has also been measured in the peripheral blood in the cubital vein outside migraine attacks [103,104]. Some authors did not confirm these data; in fact, one research group did not find an elevated CGRP level by measuring it in the external jugular venous blood during migraine without aura attack [105]. Other authors reported a significant decrease in the CGRP level in the cubital venous plasma during migraine attacks without aura as compared with the level outside the attack period [106]. The short half-life of the peptide might explain the differences observed about plasmatic CGRP levels measured from the external jugular and the cubital vein [106]. Furthermore, it was been shown that the levels of CGRP that were revealed in the saliva were significantly elevated in the inter-critical phase in migraine subjects as compared with controls [107]. In conclusion, CGRP seems to play a crucial role in the pathophysiologic mechanisms of migraine, influencing the peripheral and central sensitization of the TS and the neurogenic inflammation. Thus, it could be a possible biomarker of migraine headache.

It has also been reported that anti-CGRP antibodies do not avoid the dilatation induced by cortical spreading depression [108] and that active CGRP receptors are not expressed on the human mast cells that, furthermore, they do not manifest a CGRP response [109].

The theory of neurogenic inflammation is not sufficient to explain the relationship between CGRP and migraine attack. Lars Edvinsson et al. suggested investigating the role of trigeminal excitability in the context of sensation of pain in which CGRP takes part.

CGRP is contained in neurons of the trigeminal ganglion that emit C-fibers with intracranial and extracranial distribution. The CGRP receptor is CLR/RAMP1 (calcitonin receptor-like receptor/ receptor activity-modifying protein 1) that presents a wide distribution in the trigeminal vascular system; CGRP binds also the amylin receptor or CTR/RAMP1 (calcitonin core receptor/receptor-activity modifying protein 1), which is another target of antimigraine treatment, especially for rimegepant. Further targets of the CGRP signaling system are satellite glial cells, middle meningeal artery, and Aδ neurons [93,110,111].

Vasoactive intestinal polypeptide (VIP) is a neuropeptide composed of 28 amino acids, and it is part of the secretin/glucagon/VIP superfamily of neuropeptides. It carries out its action through vasoactive intestinal polypeptide receptors 1 and 2 (VPAC1 and VPAC2), which belong to the family of seven transmembrane G protein-coupled receptors [112,113].

Goasby et al., in 1990, showed that the plasmatic level of VIP was elevated in a clinical study including patients with spontaneous migraine attacks [96].

Various recent studies demonstrated correlation with higher VIP levels and migraine. Cernuda-Morollon et al. demonstrated that VIP levels were increased in chronic and episodic migraine patients, in the attack-free period, but not in controls subjects.

Another study analyzed the VIP levels in the peripheral blood in the interictal phase in chronic migraine, reporting that they were higher than in controls [114]. Recently, it has been shown that the administration of onabotulinumtoxin type A is useful in chronic migraine [115] and that it is possible to use VIP as a potential predictor of onabotulinumtoxin type A responders [114].

Some studies have shown the influence of some drugs used in the treatment of migraine, in particular triptans, on the VIP levels and consequently their effectiveness in the reduction of pain in migraine. For example, some authors showed how rizatriptan administration leads to a significant reduction of VIP levels measured in external jugular venous blood during spontaneous migraine attacks [116]. Other studies have brought to evidence that the use of sumatriptan reduce the salivary VIP level, which is elevated in interictal phase in patients with migraine [107]. These analysis suggest a role of the parasympathetic system in the origin of a migraine attack. This hypothesis is supported by the presence of some cranial autonomic parasympathetic symptoms, such as rhinorrhoea, lacrimation, and eyelid edema [117,118]. Another debated VIP action is the role of VIP-induced vasodilatation on the craniocervical vasculature in the induction of a migraine attack. According to Rahmann et al., a vasodilatory effect on the superficial temporal artery induced by VIP infusion was observed, but none of the subjects reported migraine attacks. It was described that VIP acted as a potent vasodilator, but the VIP infusion induced marked dilatation only of the extracranial arteries and in the craniocervical vasculature [39]. Instead, it did not play any role in the intracranial arteries in female migraineurs without aura. Therefore, only a part (18%) of the migraine patients reported migraine-like attacks [119]. In conclusion, VIP seems to act like a strong vasodilator but with a low influence on the onset of a migraine attack.

Pituitary adenylate cyclase-activating polypeptide (PACAP) is an important biologically active neuropeptide, which was isolated for the first time more than 25 years ago [120].

It is a potent stimulator of adenylate cyclase, and its gene is localized on chromosome 18 [121].

PACAP exists in two forms: PACAP-38, which is a more dominant 38-amino-acid-containing form, and PACAP-27, a C-truncated form [122,123].

PACAP is structurally and functionally similar to VIP; in fact, both forms can bind to specific PACAP (PAC1) receptors and also VIP receptors [120,123,124].

PACAP receptors are diffused in the sensory trigeminal ganglia (TRIG) and the parasympathetic sphenopalatine ganglia (SPG) [84,125,126,127].

Moreover, they are closely related to the vascular smooth muscles and are present at different levels of the CNS and PNS [128,129,130].

High concentrations of PACAP-38 have been detected among others in the human trigeminal nucleus caudalis TNC and the locus coeruleus (LC), with moderate PACAP expression in the raphe nuclei, the periaqueductal grey matter (PAG), the thalamus, and the spinal trigeminal nucleus [131,132,133].

PACAP binding sites have been identified in the cortex, the thalamus, the hypothalamus, the brainstem, the TRIG, the human mast cells, the middle cerebral arteries (MCAs), and the middle meningeal arteries (MMAs) [134,135,136].

Some data suggest that an antimigraine action may be mediated by the MrgX2 receptor (Mas-related G protein-coupled receptor member X2).

An Anti-PACAP antibody is under development. The findings from this trial will give further clarifications about the role of PACAP in migraine pathophysiology [137].

A study by Amin et al. showed that PACAP induced migraine in susceptible subjects when administered by systemic route. Another study reported high concentrations of PACAP in a group of patients during a migraine attack. PACAP, similar to CGRP, may constitute a therapeutic target [135].

NPY is a marker of the sympathetic nerve endings composed of 36-amino acids. It exercises long-lasting vasoconstrictor properties, and it also has a crucial role in the control of the cerebral circulation [138,139,140]. In the sympathetic nerve terminals, NPY is costored and coreleased with norepinephrine [141,142]. According to some immunohistochemical investigations, the locus coeruleus acts as a “migraine generator”, containing the C-terminal flanking peptide of NPY immunoreactivity in the neurons. The LC influences TNC by sending noradrenergic-containing nerve fibers [82]. NPY-IR (immunoreactive) nerve fibers are widely diffused; in fact, they innervate the cerebral arteries, pial blood vessels, and cerebral dura mater [143]. Several data suggest that NPY plays a role in the pathophysiologic mechanism of migraine with aura. In young migraineurs with aura, the levels of NPY measured in the plasma were increased during attacks, but they were found reduced in the interictal period [144].

A research group reported that the NPY immunoreactivity was higher in patients with migraine during attacks in the CSF. After lumbar puncture, the same results were not reported in controls [145]. However, other evidence did not observe an NPY immunoreactivity elevation in the suboccipital CSF or plasma during attacks and attack-free periods of patients with migraine without aura [106]. In conclusion, NPY is a good marker of the intracranial sympathetic innervation, but this role in the pathomechanism of migraine pain is still not clear.

Substance P (SP) is composed of 11 amino acids, and it binds the neurokinin 1 (NK1) receptor. Substance P belongs to the tachykinin neuropeptide family [146,147]. An important expression of SP was registered in the trigeminal sensory nerve fibers [148]. In brainstem nuclei called “migraine generators”, in the TNC and in the dorsal horns at the spinal C2 level, a wide distribution of SP-IR nerve fibers was observed [82,132]. In both PNS and CNS, substance P has a key role in pain. Furthermore, SP is involved in nociceptive conduction in TNC. When the trigeminovascular system is activated, SP induces neuroinflammation with plasma protein extravasation and vasodilatation in the cerebral dura mater [149]. This process is blocked by the selective NK1 receptor antagonists [148,150].

The NK1 receptor antagonists could also inhibit the activation of the second-order neurons in the TNC after electrical stimulation of the TRIG [150]. In preclinical studies, it was observed that stimulation of the peripheral branch of the TRIG leads to an elevation of CGRP and VIP, but not NPY and SP [67]. In the cranial venous outflow, higher SP levels during spontaneous migraine attacks were not detected [96,151]. Instead, it was observed that the salivary SP immunoreactivity was increased during spontaneous migraine attacks without aura, but not in control subjects [152]. Other evidence confirmed the correlation between SP and migraine pain; in fact, in chronic migraine subjects, SP levels in the plasma and saliva were higher than those in control subjects, and high levels were associated with pain intensity. However, it is clear that SP has a potent plasma protein extravasation effect, but this role is not clearly defined in the pathogenesis of migraine; thus more studies are needed.

Neuropeptide somatostatin (SST) (previously termed somatotropin release-inhibiting factor) is involved in the regulation of the neuroendocrine system. It acts as an inhibitor of the secretion of growth hormone, insulin, glucagon, thyrotropin-releasing hormone, cholecystokinin, gastrin, secretin, motilin, calcitonin, and parathyroid hormone [153,154]. SST has a precursor molecule, prepro-SST, consisting in 116 amino acids. This precursor molecule undergoes cleavage to give origin to two forms, SST-14 (14 amino acids) and SST-28 (28 amino acids) [155]. The SST-containing neurons are distributed in the cerebral cortex, hypothalamus, hippocampus, brainstem, and spinal cord [156]. Hannon et al. described six different SST receptor subtypes belonging in the G protein-coupled receptor family [153,156,157]. In a preclinical animal study, it was observed that the administration of cyclo-SST to the posterior hypothalamic area of rat blocked SST receptors exerted an antinociceptive effect on the dural electrical and facial thermal inputs in the TNC [158]. The SST immunoreactivity level, which was measured in cerebral spinal fluid obtained by lumbar puncture in chronic migraine patients, was lower than that in controls [159]. Recent evidence that was reported by a double-blind parallel group trial showed that the treatment of migraine attacks with the subcutaneous administration of a long-acting SST analogue (octreotide, SMS 201–995) induced a significant reduction of headache pain [160]. However, a clinical study investigated the effect of the intravenous infusion of SST in patients with or without aura. The SST administration did not lead to immediate or delayed migrainelike headaches in migraineurs or control subjects [161]. Actually, other preclinical and clinical studies are needed to clarify the involvement of SST in the pathomechanisms of migraine.

NOP (orphanin FQ) is an opioid-related peptide that binds to the orphan-like receptor 1, a member of the opioid receptor family (nowadays termed the NOP1 receptor) [162,163]. It is composed of 17 amino acids. The NOP1 receptor is expressed in various regions of CNS, such as the brainstem, the hypothalamus, and the dorsal horn of the spinal cord [164,165]. NOP exercises several effects in the CNS; it has algesic, hyperalgesic, and analgesic properties, while it has an antinociceptive action in the PNS [166,167]. NOP-ir fibers of trigeminal origin were detected in the dorsal horn of the cervical spinal cord [168]. The NOP-ir neurons also contain CGRP and PACAP [125]. NOP1 receptors were not detected in the human intracranial arteries (basilar and MCAs), and NOP immunoreactivity was not expressed in the human extracranial temporal artery [125]. NOP1 receptor is expressed in some CNS structures connected with the origin of migraine, such as the TNC, LC, PAG, raphe nuclei, thalamus, and sensory [166]. Trigeminal sensory nerve fibers innervate meningeal vasculature. Their activation can result in neurogenic inflammation consequently with migraine pain [39,151].

A clinical study revealed that plasma NOP level was lower in migraine patients without aura during the headache-free period than in controls and that the level correlated with the attack frequency [166].

Thus, the action of NOP on the NOP1 receptor could be involved in the regulation of the vasomotor response of the cerebral dura mater and thus could influence the release of the neuropeptide from the trigeminal sensory nerve terminals.

The term Oxs indicates two molecules, orexin A (OXA) (33 amino acids) and orexin B (OXB) (28 amino acids) synthesized in the lateral, posterior, and paraventricular nuclei of the hypothalamus. These two orexins derive from a precursor, the prepro-OX (130 amino acids) [169,170]. OXA selectively binds to the OX1 receptor (OX1R), while both OXA and OXB are bound to OX2R [169,171]. OX-containing neurons project to different areas of the brain, namely, cerebral cortex, cingulate cortex, paraventricular thalamic nuclei, and “migraine generators”, stimulating nociceptive transmission. In particular, in migraine-related structures, OX-containing neuron activation is highest during wakefulness and inhibited during sleep [169,172]. OX1R is selectively expressed in the LC, while OX2R is expressed in the NRM [172]. Among Oxs actions, there is the ability to modulate the TS [158]. Furthermore Oxs functions can attenuate neurogenic dural vasodilatation via OX1R activation.

A research group investigated the effects of simultaneous antagonism of OX1R and OX2R with dual OX receptor antagonist-12 (DORA-12). This study observed that the injection of complete Freund’s adjuvant to the temporomandibular joint in a rat inhibited trigeminal sensory neuronal activation in the TRIG [100]. However, a randomized double-blind placebo-controlled pilot trial showed that an OX receptor antagonist (filorexant, 10 mg nightly) was not useful in migraine prophylaxis [173].

Overall, the OXergic hypothalamic activation may be connected with the pathomechanism of migraine through OX1R and OX2R modulation of the nociceptive transmission in the TS.

## 4. Shared Environmental Factors and Stress

A wide variety of triggers are responsible for the onset of migraine episodes. In effect, numerous studies confirmed that stress, auditory stimuli, fatigue, fasting, and menstruation are among the primary triggers of migraine [174].

It is unclear which triggers are most responsible for the onset of migraine with or without aura. It is also unclear which triggers are specific for migraine patients and which for tension-type headache patients.

Women develop more migraine episodes than men. Menstruation is the most significant risk for migraine, with an increase in migraine prevalence and persistence by up to 96% [175]. In addition, migraine attacks during menstruation can be considerable and severe, and these episodes are associated with a reduced response to triptan therapy [176].

Estrogen withdrawal should be considered among the leading causes of migraine onset during menstrual period. Furthermore, migraine episodes often occur before menstruation or on the first day. However, in a few cases, there are migraine episodes towards the final menstrual cycle period, which may be correlated with the onset of anemia [177].

Another factor responsible for migraine episodes is the climate. We know that high altitudes are the leading causes of headaches, especially following rapid ascents [178]. Furthermore, another factor that may affect migraines is humidity and the gravity of environmental pollution. Patients living in extreme latitudes, such as the Arctic Circle, may develop migraines due to the seasons and the variability of sunlight [179]. The intensity of migraines can be aggravated by visual, acoustic, and olfactory stimuli, which may cause migraines in vulnerable individuals.

Several visual triggers can cause migraine [180]. Stress is one of the leading causes of migraines in the general population. Many studies demonstrated a link between chronic stress, pain, migraine, and catastrophic thinking [174,181]. Poor-quality sleep is one of the stressors responsible for migraine, so sleep can help cure an attack in many migraine patients [182]. By evaluating the lifestyle of patients with migraine, it was found that improper diet, alcohol, and obesity could promote migraine attacks. The main components of a complete diet include carbohydrates, proteins, fats, vitamins, and ions. It is unclear whether these dietary factors prevent or cause headache attacks [183,184,185]. Both obesity and migraine are associated with a high risk of activating inflammatory processes. For this reason, they may be mainly involved in the genesis of migraine pain [186].

The literature shows that the level of CGRP is increased in the plasma in both obese adults and migraine patients [187,188].

Different types of diets exist to relieve migraines. The ketogenic diet, for example, seems to improve mitochondrial activity and energy metabolism, which is why it seems to have a neutral protective role [189]. In addition, the diet acts on serotonin and reduces the calcitonin gene-related peptide (CGRP). It also inhibits the neuroinflammatory mechanisms. Considering the close correlation between obesity and diabetes, it is also possible to hypothesize that prescription of a low glycemic index diet may be promising in controlling headache through the attenuation of the inflammatory state. Additionally, obesity and headaches, including migraines, could be linked through mechanisms such as inflammation and impaired hypothalamic function. Therefore, applying dietary weight-loss strategies can also improve headaches/migraines. Furthermore, a balanced intake of essential fatty acids, omega-6 and omega-3, could be a fundamental nutritional intervention that could improve migraines. Elimination diets appear to be aimed at migraine patients with food sensitivity and are effective in preventing migraine. Taken together, dietary strategies that could be considered effective in migraine prophylaxis include weight-loss diets in obese patients with headaches, ketogenic and low-calorie diets, reduced omega-6 intake, and increased fatty acids [189].

## 5. Advances in the Genetic Basis of Migraine

Migraine is a complex polygenic genetic disease, with an estimated inheritance of up to 50% [190]. Considering its complexity, it seems that there is a genetic basis behind the development of migraines.

Genetic studies are starting to uncover genes and loci associated with migraines.

For the classic forms of migraine, twin studies showed multifactorial inheritance at the base of migraine [191]. Twin studies documented that monozygotic twins (MZ) showed approximately double the concordance rates compared with dizygotic twins (DZ) (MZ: 32%–48%; DZ: 12%–31%; Kallela, 2000, p. 9) for the development of migraine, with 42% inheritance [192,193,194]. An environmental impact is also suggested by the absence of a 100% concordance rate in the MZ twins [193].

The GenomEUtwin project is interesting, which studied the gene loci involved in the development of migraine. This study, referring to the International Headache Society (IHS) diagnostic criteria, sought to identify susceptible loci and genes responsible for the development of migraine with and without aura. These criteria resulted in a comprehensive and valuable outcome in clinical practice [195,196], but they are not perfect [197]. These IHS criteria helped research migraine and the international diagnosis of migraine, comparing pairs of migraine twins in Australia, Denmark, Finland, the Netherlands, Sweden, and the United Kingdom and their family members, who were subjected to different migraine questionnaires by country [193]. The study revealed differences in incidence between countries, showing that the environment and unshared genes contributed significantly to the predisposition to migraine, as demonstrated by many other studies. Furthermore, it revealed that diet or lower temperatures could protect against the manifestation of migraine in low-prevalence countries, such as Finland and Sweden. This study made it possible to fine-tune the IHS diagnostic criteria and identify genes involved in complex diseases, such as migraines.

Common migraine is mostly polygenic, characterized by polymorphisms and variants in many genes that cause these episodes to develop. Recent genome-wide association studies (GWAS) have identified numerous gene loci associated with migraine [198]. To date, only 13 genetically significant risk loci have been identified for the prevalent forms of migraine [199,200,201]. The transmission of three genes linked to ion transport, CACNA1A, ATP1A2, and SCN1A, is responsible for the onset of familial hemiplegic migraine [202,203,204]. Thanks to these studies, it would seem that the mechanisms that regulate the homeostasis of neuronal ion channels are also involved in the development of migraines. Indeed, two genes cause the disease in familial hemiplegics (FHM): ATP1A2 on chromosome 1q [203] and CACNA1A on chromosome 19p [202]. The CACNA1A gene is a brain-specific gene and encodes the a1A subunit of nerve cell calcium channels [202], and ATP1A2 encodes the a2 subunit of the Na^+^/K^+^ pump. It is interesting how both molecules are involved in ion transport. This aspect suggests that genes that code for similar cellular pathways are responsible for developing the most common forms of migraine [205]. The complexity of the genetic analysis arises from the lack of specific markers for migraines, the variety of diagnostic criteria, and the fact that it can only occur randomly in different family members. Furthermore, considering the symptoms, there appears to be a genetic heterogeneity behind migraine [206]. A recent genome-wide association meta-analysis (GWA) of 23,285 migraine cases and 95,425 controls of European origin conducted by the International Headache Genetics Consortium (IHGC) [201] identified 12 independent single nucleotide polymorphism loci (SNP). Referring to this study, Joe Kossowsky et al. [207] sought to understand whether genetic variants present in certain sections of the genome could be associated with the development of migraine with specific symptoms. The study compared a cohort of women who suffer from migraines and women who do not suffer from migraines. By analyzing single 12-loci nucleotide polymorphisms, a significant selective association for migraine characterized by specific symptoms and six new associations emerged [207]. The study of nucleotide polymorphisms also allowed us to evaluate the influence of diagnostic severity in selective associations. In fact, for example, in a meta-analysis, which included data from the Women’s Genome Health Study (WGHS), the rs11031122 polymorphism was identified. It is the first nucleotide specific for migraine with aura, and it is associated with migraine with aura and without aura [198]. However, in the case of migraine without aura, the second selective single nucleotide polymorphism was identified as rs1024905. This polymorphism maps close to the fibroblast growth factor 6 (FGF6) gene. Genes play a fundamental role in the mechanisms of cell proliferation and cell differentiation. They also play a role in angiogenesis and myogenesis. It is also essential for normal muscle regeneration [190]. Finally, another polymorphism, rs12260159, plays a relevant role. It is located in the intron of the heparanase 2 (HPSE2) gene, which is associated with an orofacial syndrome in humans to develop migraines [208]. It is interesting to note how other studies have evaluated the association between the onset of migraines and the presence of inflammatory mechanisms.

To precisely evaluate this aspect, additional studies were performed that compared the results of the GWA analysis of patients with Alzheimer’s alone with the results of the GWA analysis of patients with only MO. However, the GWA samples of patients affected by Alzheimer’s were insufficient to draw a valid conclusion regarding the genetic overlap between patients with Alzheimer’s alone and patients with Alzheimer’s and MO only. Instead, these studies highlighted how the TRPM8, UFL1, FHL5, and LRP1 genes (previously described in the GWA studies) and how two new genes, TARBP2 and NPFF, are mainly associated not only with the risk of developing migraines but also with inflammatory processes and cardiovascular diseases. Therefore, they also appear to be a possible cause of Alzheimer’s and MO [209]. 

As regards the genes TARBP2 and neuropeptide precursor of the FF-amide peptide (NPFF), these represent new candidate genes for migraine risk. TARBP2 (TAR) is an RNA-binding protein encoded by the TARBP2 gene required to form the RNA-induced silencing complex. TARBP2 binds between the swelling zone and the loop that regulates HIV-1 TAR RNA and activates HIV-1 gene expression along with the viral Tat protein. TARBP2 is also an integral component of the DICER1-containing complex. It is involved in miRNA processing [210]. Based on the findings of studies, there is no clear functional link between TARBP2 and migraine risk. The neuropeptide precursor of the FF-amide peptide (NPFF) modulates the analgesia induced by morphine. It also increases blood pressure and somatostatin secretion from the pancreas. NPFF has the function of binding the coupled receptor to the G protein, regulating the receptor’s binding and the activity of the neuropeptide hormone. In particular, the neuropeptide FF sensitizes and enhances the effect of the acid-sensitive ion channels ASIC1 and ASIC3. These are protonated channels capable of carrying Na^+^ and Ca^2+^ [211,212]. By evaluating the decrease in extracellular pH by blocking the ASIC channels, it was observed that the primary dural afferent neurons could be directly stimulated through the opening of the ASICs.

Furthermore, it caused typical pain related to migraine. This finding suggests that ASIC inhibitors may represent new frontiers for migraine therapy [212]. Moreover, the ASIC inhibitor amiloride can also inhibit cortical diffusion depression (CSD) and block trigeminal activation in vivo in animals via an ASIC1 mechanism. These results suggest that NPFF is the gene that is most associated with the risk of developing migraines.

A more recent research field is represented by RNA sequencing technology that will allow us to provide a more defined expression profile in the trigeminal vascular system through the analysis of the intrinsic molecular determinants of the pathophysiology of migraine. Among the most relevant recent molecular findings, the expression of GABAB receptors at the nodes of Ranvier may be cited in the dorsal root ganglion. The part played by axon–glia communication has been suggested, but this role in conduction needs further investigation [213,214].

With regard to genetic aspects, some single-gene mutations have been detected in rare migraine syndromes, such as familial hemiplegic migraine and monogenic vasculopathies. Genome-wide association studies have not yet provided great findings about the identification of mutations with relevant effect sizes. Two mutations of the gene encoding CK1δ (casein kinase 1 δ) have been identified in two families with migraine. This genetic alteration has also been associated with a circadian rhythm disorder (familial advanced sleep phase syndrome) [215].

Some population studies have proved an association between 38 genomic loci and migraine. This considerable number of genes and their different functions emphasize the complex role that genetics plays in the pathophysiology of migraine underling the strong likelihood that numerous genes and epigenetic factors interact in this context [198,199].

The main genes implicated in migraine are expressed in gastrointestinal tissue and the vascular system. This evidence further supports the role of vascular processes in migraine. Other genes that are associated with a higher risk of migraine were reported in a population study by Yin et al. and are related to genetically mediated hypercalcemia.

The study of genetic mechanism will give the possibility to tailor migraine treatment in relation to specific genes responsible for migraine [216,217].

Knowing which genes are also responsible for the development of migraines is therefore essential, as today we are increasingly aiming to perform personalized therapy. Indeed, Kogelman and others have showed a crucial interindividual variation in response to migraine therapy [218]. Therefore, by evaluating the genetic basis and the associated polygenic risk, it seems possible to choose the best therapeutic strategy for acute migraine attacks and prevention.

## 6. Novel Approach to Acute Treatment and Prophylaxis of Migraine

According to the data reported in the CaMEO Study, 41% of patients with chronic migraine were in the care of physicians, and just 25% of these patients got an accurate diagnosis of their migraine [219].

### 6.1. Acute Treatment

Triptans have represented the cornerstone of the acute treatment of migraine for many long years. They were introduced in the 1990s in migraine management. Triptans belong to serotonin 5-HT_1B/1D_ receptor agonists with potential vasoconstrictive effects; in fact, their extensive employment has been limited by their cardiovascular risk profile [220,221].

The efficacy of triptans as acute therapy highlights the importance of neurogenic inflammation; in fact, this class of drugs acts to prevent plasma protein extravasation and reduce the release of neuropeptides [222,223].

Triptans, which are the most used acute drugs in the treatment of migraine, cause the activation of Gi-coupled 5-HT1B/1D receptors and thus the reduction of the intracellular levels of cAMP. These effects lead to a lower excitability of unmyelinated C-fibers reducing CGRP release [224].

The 5HT1 receptors are bound by some triptans and by a new class of drugs called ditans, including LY344864 and LY334370 and showing a lower affinity for the 5-HT1B/1D receptors. Through a specific agonism on a 5-HT1F receptor, these drugs impede the extravasation of protein in the dura, and they cause an irrelevant vasoconstriction. This mechanism of action demonstrates the validity of an approach consisting in activating a 5-HT1F receptor, which is an GI protein-coupled receptor, thus preventing the release of CGRP from the trigeminal vascular system [225,226,227].

Two methods of acute treatment exist: a stratified method and phased approach. The choice of the type of treatment depends on the patient factor and the characteristics of the attachment. A stratified method is considered for patients who can distinguish and recognize their headaches, selecting the most suitable medication. It was reported that this strategy shortened the time to attack resolution and reduced headache treatment costs and the disability time [228]. A phased approach is beneficial. It is necessary to take nonsteroidal anti-inflammatory drugs (NSAIDs) as quickly as possible before the migraine peak and then take a triptan or dihydroergotamine to reduce the extent of the migraine. This approach was reported to defer headache resolution. American Headache Society guidelines recommend acetaminophen, aspirin, and NSAIDs for mild attacks as therapy. In the case of moderate to severe attacks, triptans and dihydroergotamine (DHE) are recommended. In comparison with DHE, triptans are characterized by better tolerability and efficacy. Nasal, injectable, and subcutaneous formulations exist and yield more effective results in those patients with a severe rapid onset of headache associated with nausea and vomiting. A second triptan dose should be taken if the administration of a triptan results in an initial relief that is, however, followed by a recurrent, more severe attack within 24 h. On the other hand, patients who present a partial or inconsistent response after taking a triptan should consider taking sumatriptan associated with NSAIDs [229,230]. The treatments reported in the guidelines may not benefit all patients and are sometimes characterized by contraindications related to coexisting disorders. Contraindications to the use of triptans and DHE are coronary heart disease, peripheral vascular disease, and conditions such as migraine with brainstem aura. In addition, subjects with a history of peptic ulcer disease and gastrointestinal bleeding should avoid taking NSAIDs; moreover, the latter should be used with caution in migraineurs with an increased cardiovascular risk profile. Another contraindication concerns zolmitriptan, rizatriptan, and sumatriptan. They must not be taken within 14 days of using monoamine oxidase inhibitors [229].

It is necessary to introduce new, safer, and more effective treatments and develop innovative therapeutic options in consideration of side effects and poor tolerability (Table 1).

New emerging therapies consist of ditans, which are agonists of the serotonin 5-HT1F receptor. The first drug to be approved of this class was lasmiditan, which acts on the 5-HT1F receptor and reduces the extravasation of proteins in the trigeminal ganglion. Since 5-HT1F is not expressed in the vascular system, the effects of lasmiditan are not vascular. Furthermore, this drug does not cause vasoconstriction as ditans inhibit nociceptive trigeminocervical transmission [231,232,233].

Dosages of lasmiditan 100 and 200 mg were studied in SAMURAI. This study is a randomized, phase 3, placebo-controlled trial. This study reported 59% pain relief at 2 h compared with 43% in the placebo group. Another finding was that 41% of the subjects experienced alleviation of the main disturbing symptoms with lasmiditan 100 and 200 mg. This percentage was 31 in the placebos [226].

The SPARTAN trial showed positive effects of lasmiditan 50, 100, 20 mg in terms of relief from pain at 2 h and comfort from disabling symptoms [234].

The SPARTAN trial enrolled 2583 participants randomized into 1 of 7 groups (lasmiditan 50 mg plus placebo, lasmiditan 100 mg plus placebo, lasmiditan 200 mg plus placebo, 2 lasmiditan 50 mg doses, 2 lasmiditan 100 mg doses, 2 lasmiditan 200 mg doses, 2 placebo tablets). All 3 lasmiditan groups reached a greater statistical significance than placebo for the primary endpoints percentage of headache-free participants 2 h after the dose and percentage MBS-free 2 h after the dose [235].

The GLADIATOR trial was recently completed. It enrolled subjects from the SAMURAI and the SPARTAN trials; it assessed the efficacy of the second dose of Lasmiditan 100 or 200 mg for recurring migraine attacks or rescued acute migraine [236].

The 5-HT1F receptor is a nonvascular serotoninergic receptor that is not targeted by the triptans. It does not cause vasoconstriction; thus, it may constitute a valid alternative for patients with cardiovascular disease.

The ditans activate 5-HT1F receptors blocking the trigeminal neurons’ activation and inhibiting the acute migraine pathway. These main features make triptans preferable to triptans [237,238].

In recent years, numerous studies have shown the role of CGRP in migraine attacks. These studies have allowed the development of a new class of drugs that are CGRP receptor antagonists and gepants. The CGRP receptor is expressed in both peripheral and central neurons. Antagonists modulate thalamic activity in response to the trigeminal nociceptive signal. It is a new class of drugs that includes small molecules, such as ubrogepant and rimegepant, which block the release of CGRP as part of the migraine pathway. These molecules thus inhibit neurogenic inflammation and vasodilation [239].

Most trials on gepants investigated their usage principally on acute migraine treatment.

Ubrogepant is a CGRP antagonist receptor approved by the FDA for acute migraine therapy with or without aura. It is orally administered. Two trials, ACHIEVE I and ACHIEVE II, reported positive results concerning the primary endpoints of relief from pain for 2 h and resolution of main disturbing symptoms for both doses, ubrogepant 50 and 100 mg. ACHIEVE I randomized 1327 patients to placebo, ubrogepant 50 mg, and ubrogepant 100 mg [240].

This study reported that both doses of ubrogepant achieved statistical significance for the coprimary endpoint of the percentage of individuals who were free from pain at 2 h after the dose [241].

ACHIEVE II is a multicenter, placebo-controlled double-blind, randomized trial that evaluated the efficacy of ubrogepant 25 and 50 mg versus placebo in terms of freedom from pain 2 h after the dose in subjects with moderate to severe migraine. Both amounts were found to gain statistical significance when compared with placebo. Furthermore, the main results of ubrogepant 50 mg were the reduction of bothersome symptoms at 2 h after the dose and resolution of the symptoms of photophobia and phonophobia at 2 h after the dose. They also documented significant pain relief and pain free for 2 to 24 h.

ACHIEVE II randomized 1355 patients to reduced doses of 25 and 50 mg; both doses were associated with relevant results in producing relief from pain for 2 h; moreover, the 50 mg dose resulted in the absence of troubling mental symptoms [242].

Rimegepant is another CGRP receptor with oral administration. It is currently investigated in a phase 2 trial for migraine prevention. In July 2019, the results of the first studies on rimegepant reported its efficacy in the acute treatment of migraine compared with placebo [243].

In particular, it was reported that 1 dose of rimegepant achieved 2 primary endpoints of freedom of MBS at 2 h after the dose and from, pain and its effects increased through the first 8 h with maintenance until 48 h [244].

Safety and efficacy were comparable between the group of subjects receiving rimegepant and the placebo group. However, nausea and urinary tract infections were among the most common side effects. Rimegepant will be the first drug of this new class to provide efficacy in the acute and preventive treatment of migraine.

Atonement is an oral gepant that was conceived to prevent migraine. The first results of the studies investigating atogepant were encouraging in particular. They reported a decrease of 4 monthly migraine days; another trial on its usage for prevention is ongoing [245], [246] (p. 3).

### 6.2. Prophylaxis

Prevention consists of treatment that should reduce the severity and duration of attacks and the frequency of migraine. Preventive medicines are indicated for patients suffering from more than six headache days for the month for patients with recurring migraine attacks causing relevant disability, with contraindications for acute therapies and other special conditions [247,248].

Migraine prophylaxis medications approved by FDA include beta-blockers, antiepileptic drugs, calcium channel blockers, and tricyclic antidepressants; however, current guidelines lack evidence that establishes a first therapeutic option over another (Table 2). The choice of preventive therapy is thus based on the patient’s comorbidities and the risk profiles of the subject, and the therapeutic efficacy takes approximately 6 months to be reached [249,250].

Currently, several different medications initially assigned for other indications are used for migraine prevention in clinical practice. These drugs exert their effects and are effective through various mechanisms of action. They suppress excitatory nerve signaling via sodium and calcium receptors. They also allow for blocking cortical diffusion depression, reducing neuronal sensitization, promoting GABAergic inhibition, and decreasing CGRP levels. Preventive treatment is strongly recommended for those patients affected by frequent severe attacks.

Beta-blockers that are efficacious in migraine prevention are propranolol (80–240 mg/die), timolol (20–30 mg/die), metoprolol (200 mg/die), bisoprolol (5 mg/die), as reported in clinical studies [251,252].

Beta-blockers have been reported to be useful for migraine prevention. They work by inhibiting beta 1-mediated effects and block norepinephrine release and tyrosine hydroxylase activity. Furthermore, β-blockers exert an action mediated by GABA, which modulates the firing rate of neurons of the periaqueductal grey matter. Some studies also reported that some beta-blockers influence the serotoninergic system by blocking 5-HT2C and 5-HT2B receptors [253].

Other effects have been shown for propranolol: the inhibition of nitric oxide production and kainite-induced currents. Propranolol and metoprolol reduce the VEP amplitude in migraine patients: this effect may be associated with beneficial response in the preventive treatment. In the light of this finding, it has been suggested that this class of drug acts on the excitability of the visual system. Additionally, the effects on auditory evoked cortical potentials have been reported and associated with a reduction in the frequency of migraine attacks [252,254,255].

In short, neurophysiological studies report that beta-blockers exert a preventive action in the cortical inflammation processes of migraine. These studies have also shown that they act at the level of cortical excitability. They also influence the systems regulated by norepinephrine and serotonin. Animal experimental studies support the idea that cortical spreading depression may represent another target for this pharmacological class [182,256,257].

Several antiepileptic drugs are suitable for the preventive treatment of migraine. In particular, topiramate and valproate report the best clinical evidence in migraine prevention studies. Topiramate acts by multiple mechanisms of action, such as blocking various channels, such as high-voltage-activated L-type calcium channels and voltage-gated sodium channels. It also inhibits glutamate-mediated excitatory neurotransmission. It also inhibits the activity of carbonic anhydrase and promotes GABA-A-mediated inhibition. It also reduces the release of CGRP from trigeminal neurons [258]. However, it is currently unknown what the main primary mechanism is providing topiramate with its efficacy in preventing migraine.

Valproate’s beneficial effects in reducing migraine frequency are attributable to numerous mechanisms, such as enhancement of gabaergic inhibition, the block of excitatory ion channels. In particular, it blocks voltage-dependent sodium and low-threshold T-type calcium channels [259].

Valproate retains the protein kinase C, which regulates glutamatergic neurotransmission. Recent findings suggest a different mechanism of action: inhibition of the NF-kB (nuclear factor kappa-light-chain-enhancer) pathway and downregulation of CGRP expression [260,261].

Among calcium antagonists that have been used for migraine prevention since the 1980s, flunarizine is the most used compound. This is because it acts as a nonselective calcium antagonist, for which the mechanism of blocking voltage-gated sodium channels has been reported. Thanks to these two actions, flunarizine decreases neuronal excitability and stabilizes cortical hyperexcitability. In addition, other effects have been reported as the reduction of the number and duration of cortical spreading depression (CSD) waves and the mitochondrial injury induced by CSD.

Verapamil and cinnarizine are off-label drugs for migraine therapy. They are indicated in refractory migraine forms or cases of unavailability of flunarizine [237,238,262].

The results of more than three placebo-controlled trials have shown the superiority of amitriptyline (a tricyclic antidepressant) and fluoxetine (a selective serotonin reuptake inhibitor) over placebo: they are used for migraine preventive treatment. This efficacy has been reported for doses lower than those recommended for depression. Other compounds of this class are less effective for migraine preventions: some evidence has been reported that venlafaxine, a selective serotonin–norepinephrine reuptake inhibitor and other tricyclics. It has been suggested that the efficacy of amitriptyline is partly due to numerous channel and receptor (sodium channel blocker) effects and its action on alpha-2. In this regard, blocking the alpha2 adrenergic receptors prevents the antinociceptive effect (t of amitriptyline) and could inhibit the alpha-2 adrenergic receptors.

As with other preventive drugs, it is poorly known which is the crucial mechanism mainly responsible for the positive effects of amitriptyline.

Amitriptyline is a serotonin and norepinephrine reuptake pump inhibitor. It appears to promote inhibition and thus influence the pain control mechanisms that descend from the brain stem to the caudal trigeminal nucleus and spinal cord. Fluoxetine is a selective blocker of serotonin reuptake from the synaptic cleft, leading to increased serotonin levels; additionally, fluoxetine acts as a reversible 5-HT2C receptor blocker, which partly explains its efficacious migraine prevention. A polymorphism of the 5-HT2C gene has been associated with the modulation of migraine susceptibility. Moreover, this action is typical of previous antiheadache drugs, such as methysergide [263,264].

Onabotulinumtoxin A is a protein complex produced by *Clostridium botulinum*, a Gram-positive anaerobic bacterium. Botulinum toxin includes seven serotypes. Serotype A has been used for human therapeutic purposes since the 1980s, when it was injected into extraocular muscles. In addition, it has been used as an alternative treatment to strabismus surgery [265,266].

The two phase III trials of the PREEMPT (REsearch Evaluating Migraine Prophylaxis Therapy) program reported the efficacy of onabotulinumtoxin A for chronic migraine: the administered dose was 155 U up to a maximum amount of 195 U. According to the results of these studies, the treatment consists of the injection of fixed amounts of BoNT-A (5 units per site for a total of 155 teams) at fixed sites, which are the following muscles: procerus, corrugator, frontalis, temporalis, occipitalis, cervical paraspinal muscle, and trapezius. The findings were: reduction in headache and migraine days, moderate/severe headache days, and improvement in psychological distress and health-related quality of life [115].

The detailed mechanism of action through which BoNT-A produces positive effects in chronic migraine patients is not known. Currently, the central hypothesis is that the toxin exerts its impact through the direct inhibition of peripheral sensitization. It decreases exocytosis and the activity of the neuropeptide and neurotransmitters of peripheral sensory neurons. This effect indirectly produces a reduced central sensitization that plays a crucial role in chronic migraine [267].

Another involved process concerns several intracellular events, such as fusing vesicles containing neuropeptides and neurotransmitters with the nerve cell membrane. VAMP/synaptobrevin proteins interact with proteins expressed on the internal membrane surface SNAP-25 (synaptosomal-associated protein). This complex is called SNARE complex (soluble N-ethylmaleimide-sensitive factor attachment protein receptor) [268,269].

Onabotulinumtoxin A interferes with intraneuronal vesicular fusion, enters neurons by endocytosis, inhibits neuropeptide release, and causes receptor downregulation. The primary main neurotransmitters whose the toxin release seems to inhibit are GABA, catecholamines, dopamine, aspartate, and monoamine neurotransmitters. All these signaling molecules take part in the pathogenesis of chronic migraine [270,271].

Other compounds that are currently used for migraine prevention include ACE (angiotensin-converting enzyme) inhibitors and sartans (angiotensin receptor blocker (ARB)).

Among ARBs, candesartan showed beneficial effects at a dose of 16 mg per day in migraine prevention; in particular, it presented a similar efficacy to propranolol [272].

A small, double-bind placebo-controlled crossover trial showed that the ACE inhibitor lisinopril was significantly efficacious at a dose of 10 mg twice per day [273].

The rationale for using ARB and ACEI agents emerges from the knowledge that components of the cerebral RAS (renin–angiotensin system) play a role in migraine pathogenesis. It has mainly been reported that brain RAS can affect the astrocytic activity and endothelial cell functions. In addition, angiotensin affects sympathetic secretion and promotes the release of medullary catecholamines from the adrenal gland.

Angiotensin acts as a traditional circulating hormone and a local signaling molecule involved in neuronal function by working as a component of the cerebral tissue renin–angiotensin system.

Angiotensin II type 2 receptors are also expressed on neurons, endothelial cells, and astrocytes. They are mainly expressed in cerebral areas, regulating sensory processing [274,275].

Furthermore, it has been reported that ACE inhibitors can modulate the endogenous opioid system, vasoreactivity, and sympathetic tone and favor the removal of some proinflammatory substances, such as enkephalin, bradykinin, and substance P [276,277].

Other mechanisms of action that can explain the positive effects of these two classes of drugs are linked to different activities of angiotensin II: modulation of the potassium channel, cerebrovascular flow, increase in dopamine concentration, and activation of NF-kB, which leads to an increased expression of inducible nitric oxide synthase [278,279].

Feverfew is a plant belonging to the Asteraceae family. It has several properties, making it efficacious in migraine prophylaxis. Some evidence suggests that parthenolide, an active ingredient of feverfew, attenuates Fos-mediated activation of the nucleus trigeminal caudalis, which takes part in migraine pathogenesis, and it shows an agonist activity in TRPA1 channels, involved in migraine pathogenesis too [280,281], [282] (p. 1).

Although some double-bind placebo-controlled studies reported the efficacy of this herbal remedy in the preventive treatment of migraine, it was frequently associated with adverse events, such as loss of taste, gastrointestinal symptoms, and ulcers of the mouth [283,284,285].

The antinociceptive and anti-inflammatory effects of feverfew extracts may partly explain its influence on NOS expression inducible in the degradation of the NF-kB inhibitory protein. Furthermore, feverfew has been reported to modulate 5-HT neurotransmission through the effects of antagonists on 5-HT2B and 5-HT2A receptors, which are involved in brain plasticity associated with migraine [233,286].

Butterbur, or *Petasites hybridus*, root is a herbal supplement historically used for medicinal purposes. It acts through anti-inflammatory action, such as inhibiting cyclooxygenase 2 and blocking Ca^2+^ channels by petasites that are coactive components. However, recent findings reported moderate antimigraine effects [287,288,289].

Among metabolic treatments, significant, exciting findings reported in clinical trials concern riboflavin, coenzyme Q10, lipoic acid, L-carnitine, and ketogenic diet. Riboflavin is a vitamin involved in mechanisms of oxidation–reduction during several cellular energy processes. MR spectroscopy studies allowed us to point out a decreased potential of mitochondrial phosphorylation and reduced ATP levels in the cortex of migraineurs [290,291].

As reported in a placebo-controlled double-blind trial, riboflavin showed significant efficacy in migraine prevention. Positive results were also reported in an open-label study and in a trial where riboflavin was compared with propranolol [292,293].

It was the first metabolic enhancer evaluated in migraine. A study by Boehnke reported the effects of riboflavin in migraine prevention, showing a reduction in migraine severity and frequency of the attacks and a significant 50% responder rate of 80%. A multicenter, randomized placebo-controlled clinical trial reported that riboflavin 400 mg QD reduced attack frequency compared with placebo, a 50% responder rate of 59% versus 15% for placebo [294,295].

Some patients are responders to preventive treatment with riboflavin; this condition may be partly attributed to the mitochondrial DNA haplogroup H associated with better oxidative phosphorylation (OXPHOS) function. On the other hand, patients who do not carry the haplo mentioned above have presented excellent response to riboflavin [296].

Some evidence supports the positive effects of other compounds, such as folic acid, folic acid combined with B6 (25 mg) and B12 (400 µg), niacin. A double-blind, randomized controlled trial investigated the effects of folic acid or folic acid combined with B6 and B12 at a daily dose of 2 mg. Its findings were a reduction of frequency and severity of migraine with aura and attenuation of migraine disability. Furthermore, the results suggested that carriers of the C allele of the methylenetetrahydrofolate reductase (MTHFR) gene variant, C677T, and the A allele of the MTRR (methionine synthase reductase) gene variant, A66G, exhibited a better response with notable clinical effects and reduced homocysteine levels [297,298].

The involvement of mitochondrial energy metabolism in the pathophysiology of migraine has led to research on the role of coenzyme Q10 or ubiquinone. Coenzyme Q10 is the complex III component of the electron transport chain. An open-label study investigated the effectiveness of the administration of CoQ10 at a daily dose of 150 mg as prophylactic treatment and reported that 61.3% of the patients that completed the study presented more than 50% of reduction in migraine days. Another result of this pilot study was a significant decrease (7.34 vs. 2.95) in mean monthly migraine days between the baseline and the third month of the subsequent randomized controlled clinical trial, which evaluated the efficacy of a double dose of CoQ10, administered as liquid suspension 100 mg t.i.d. The results supported the beneficial effects in migraine prevention in terms of reduction of headache days, frequency of migraine attack, and days with nausea. Another study reported a fascinating, exciting finding: decreased levels of CGRP and TNF-alpha in a sample of women that associated a supplementation of CoQ10 to other prophylactic treatments [299,300].

Some evidence supports the potential beneficial effects of lipoic acid, a cofactor of the Krebs cycle enzymes, in migraine. The reason that led to the investigation of lipoic acid was its involvement in regulating cerebral energy availability that was described in a case report concerning a patient with a mitochondrial disorder [301].

A placebo-controlled trial was conducted to evaluate its efficacy at a dose of 600 mg QD. However, this trial was not completed because of a limited number of patients enrolled. Nevertheless, after 3 months of treatment, the results concerning this sample of subjects showed a significant reduction of frequency and severity of the attacks. These results were not observed in the placebo group. Two further open-label trials support these encouraging results, suggesting that lipoic acid supplementation as add-on therapy to prophylactic treatment with topiramate provides a better response [292,302].

Other studies investigated the effects of L-carnitine supplementation on the prevention of migraine. L-carnitine is an amino acid derivative that plays a crucial role in producing energy by transporting fatty acids into cells’ mitochondria [303].

A recent double-blind study showed positive results on the pharmacological management of migraine through coadministration of CoQ10 and L-carnitine. A clinical trial by Esfanjiani and colleagues randomly assigned 133 migraine patients to one of these four groups: control group receiving standard prophylaxis, intervention group (magnesium oxide plus L-carnitine), magnesium oxide at a daily dose of 500 mg, and L-carnitine (500 mg/die), as add-on therapy to conventional preventive medications. Good clinical responses were reported significantly and considerably in patients receiving magnesium oxide. Conflicting results were described by Hagen et al., who did not notice benefits after administering acetyl-L-carnitine at a dose of 3 g/day for 12 weeks in comparison with the placebo group [304,305,306].

### 6.3. Diet

Diet has been considered an essentially important part of migraine. However, in the absence of proven scientific evidence, some foods are believed to represent a trigger of migraine attack; therefore, special dietary restrictions are often advised or recommended and applied by patients [307].

The ketogenic diet is a very-low-carbohydrate diet (usually <50 mg/die) combined with a relative increase in fat and protein proportions. This diet regime induces the metabolism forcefully to produce energy from lipid by free fatty acids’ mitochondrial beta-oxidation [308].

In these metabolic conditions characterized by an almost totally absent metabolism of exogenous glucose as an energy source, after a few days occurs an increase in circulating and urinary levels of endogenous ketone bodies (β-hydroxybutyric acid, acetone, acetoacetate); this process is caused by the metabolism of fatty acid from adipose tissue depots and subsequent hepatic conversion to ketones [308,309].

The potential beneficial effects of ketogenesis were previously suggested, however, in the absence of a clear explanation. The first study on the role of the ketogenic diet in migraine prevention was conducted by Di Lorenzo et al. The study was conducted in overweight women suffering from migraines and showed a significant reduction in medication intake, attack frequency, and headache days compared with the group receiving a standard low-calorie diet [310,311].

Another study compared the effects of a very-low-calorie ketogenic diet. They reported a ≥50% responder rate concerning monthly migraine days of 74.28% compared with 8.57% found in the group undergoing the second dietary regime. Moreover, these findings suggested that the therapeutic antimigraine effects of the ketogenic diet were independent of weight loss. A relevant difference in weight loss between the two regimes was not described [312].

An alternative strategy to increase ketone bodies is the supplementation of diet with exogenous ketones; these compounds influence anaplerosis, an energetic process supplying the whole of intermediates in the Krebs cycle [303].

Recent small studies investigated the efficacy of some ketone body donors as triheptanoin, reporting good response in the preventive management of migraine. However, they are characterized by gastrointestinal intolerance [313,314].

Magnesium is an essential cofactor for hundreds of human enzymes. It participates in numerous cellular processes, some of which are involved in migraine pathogenesis, regulating mitochondrial oxidative phosphorylation, nitric oxide system, and NMDA receptor function. However, conflicting findings have been reported, making limited the evidence about the efficacy of magnesium in migraine prevention, for example, for short-term prevention of menstrual migraine [315,316,317]. The indication to add magnesium supplementation in the management of migraine prophylaxis emerges from studies suggesting a deficiency of Mg^2+^ in the central nervous system of patients suffering from migraine and peripherally. However, clinical trials showed contradictory results. Some of them found oral supplementation to be effective; others reported no difference compared with placebo.

## 7. Novel Classes of Drugs

In 2018, the FDA approved three CGRP monoclonal antibodies for the preventive treatment of migraine: erenumab, fremanezumab, and galcanezumab. A subcutaneous formulation has been created, which is administered monthly or quarterly (fremanezumab 675 mg). Results from studies conducted with these drugs reported a more significant reduction in the average number of migraine days per month. The most common side effects were injection-site reaction.

Recent drug classes primarily target the CGRP pathway. These drugs include monoclonal antibodies to the CGRP pathway and gepants (small-molecule CGRP receptor antagonists). They are recommended as preventive and acute treatments, respectively [318].

Because of their significant molecular weight, it is assumed that CGRP monoclonal antibodies do not cross the blood–brain barrier, exerting thus a peripheral action on trigeminovascular structures. The main features of this class of drugs include targeted treatment, a long half-life that makes them deliverable at longer intervals, and minimal side effects. The FDA has recently approved four monoclonal antibodies: eptinezumab, erenumab, fremanezumab, galcanezumab [319,320].

In 2018, the FDA approved erenumab as first CGRP antagonist for the prevention of migraine in adults. Results from trials reported the favorable safety and good tolerability of erenumab 70 mg in chronic and episodic migraine. ARISE and STRIVE are two significant phases 3 trials that reported excellent results in the reduction in mean monthly migraine days and all secondary endpoints as a reduction of 50% in the mean number of days of consuming acute migraine-specific drugs.

ARISE was conducted in 577 patients with episodic migraine over 12 weeks.

The STRIVE trial investigated the effectiveness of erenumab 70 and 140 mg on the prophylaxis of episodic migraine in 955 subjects. Of the patients, 43.3% in the 70 mg group reported a reduction of 50% in MMD (migraine days monthly), 50% of the subjects in the 140 mg group and 26.6% of the individuals in the placebo group reported a reduction of 50% in MMD. The phase 2 trial compared erenumab 70 and 140 mg with placebo for evaluating the safety and efficacy of erenumab as a prevention treatment in patients with chronic migraine. In the 70 mg group, 40% of the patients obtained at least a 50% reduction in migraine. This percentage was 41% in the 140 mg group and 23% in the placebo group.

LIBERTY is another study including subjects who failed more than two migraine prophylactic medications (this group of patients was excluded in ARISE and STRIVE). Patients with episodic migraine were randomized to either placebo or placebo erenumab 140 mg. In the treated individuals, a response rate of 50% was observed in reducing monthly migraine days, and 14% in those receiving placebo. This study thus demonstrated the efficacy of erenumab 140 mg in this difficult-to-treat patient group [321].

The molecular form of erenumab is a fully human antibody IgG2 that targets the calcitonin-like receptor/RAMP complex. The recommended doses are 70 and 140 mg every month, administered subcutaneously. 

Constipation and injection site reactions were the most common adverse reactions that were described. In addition, in the ARISE trial, nasopharyngitis and upper respiratory tract infections were reported as frequent adverse drug reactions.

Since September 2018, fremanezumab has been approved by the FDA. It is a humanized monoclonal antibody targeting the alpha and beta of the ligand of calcitonin gene-related peptide. Its dosing schedules provide a monthly or quarterly administration frequency, with a recommended dose of 225 and 675 mg, respectively. The HALO episodic migraine prevention trial showed a reduction in mean monthly migraine days with fremanezumab. Fremanezumab 225 mg reduced this primary endpoint by 3.7 days and fremanezumab 675 mg by 3.4 days; placebo arm reported −2.2 days. Positive results were also reported in phase 3 of the HALO chronic migraine prevention trial conducted in 1130 subjects for 12 weeks. The average reduction in the number of headache days monthly was 4.6 for fremanezumab 255 mg and 4.3 for fremanezumab 675 mg and 2.5 for placebo [44,322]. 

Galcanezumab is a humanized anti-CGRP IgG4 that targets the CGRP ligand, and its route of administration is subcutaneous. The FDA approved it in September 2018. EVOLVE-1 and the EVOLVE-2 are two randomized controlled trials conducted on a sample of patients with episodic migraine evaluating the effects of galcanezumab 120 and 240 mg. The results showed a reduction in mean monthly migraine days and good safety and tolerability profile. In EVOLVE-1 t, the average decrease in monthly headache days was 4.7 in the 120 mg group, 4.6 in the 240 mg group, and 2.8 in the placebo group. In EVOLVE-2, the mean reduction in monthly headache days was 4.3 in the 120 mg group, 4.2 in the 240 mg group, and 2.3 in the placebo group. The most commonly reported adverse reactions were erythema, swelling, and pruritus at the injection site [323,324].

The REGAIN study was conducted on chronic migraine subjects and demonstrated positive findings with both dosages [325,326].

Eptinezumab is an IgG1-subtype monoclonal antibody that is administered intravenously approved by the FDA in February 2020. It is recommended at 100 or 300 mg every 3 months for preventive treatment in adult patients [327].

PROMISE-1 and PROMISE-2 are two phases 3 trials of eptinezumab: the first one was conducted in patients with episodic migraine and the second one in those with chronic migraine. Their secondary outcome measure was the probability of a migraine attack at day 1 after infusion. Both trials obtained encouraging results: in PROMISE-1, the possibility mentioned above was decreased by 45% to 53.6% in three treatment arms compared with 20.7% reported in the placebo group, in PROMISE-2, the likelihood of migraine attack at day 1 was diminished by 51% and 53% for the dosages 100 and 300 mg, respectively, and in placebos, it was 27% [328,329].

In comparison with other monoclonal antibodies, eptinezumab has a further advantage: its rapid onset of efficacy, which makes it applicable in the acute treatment of migraine. The most commonly reported adverse reactions were upper respiratory tract infections and urinary tract infections.

## 8. Neuromodulation

A nonpharmacological strategy for migraine treatment is noninvasive neuromodulation. It is a method that modulates pain without the unwanted side effects associated with most of the available drugs. Some of these modalities were first studied in an animal model in which the employment of a fluctuating magnetic wave was used to inhibit cortical spreading depression and modify the thalamocortical signaling network [330].

Single-pulse transcranial magnetic stimulation was evaluated in a sham-controlled trial including 164 patients. The results were significant in terms of pain-free response rate at 2 h, which was 39% in the stem group compared with 22% in the sham group. ESPOUSE, an open-label trial, investigated the effects of sTMS in the prophylactic treatment of migraine in 132 patients. It reported a prominent decrease of 2.75 headache days. sTMS was approved by the FDA for acute and preventive medicine. The most common side effects reported were tinnitus, dizziness, and lightheadedness [331,332].

## 9. Conclusions

Migraine is a serious pathological condition that affects over 15% of the world population [333].

Migraine is a complex polygenic genetic disease with an estimated inheritance of up to 50% [190]. Considering its complexity, there appears to be a genetic basis behind the development of migraine. Knowing which genes are responsible for the development of migraines is therefore essential, as today we increasingly aim to perform personalized therapy. Knowing the neurophysiological mechanisms and compromised neurotransmitters in inflammatory processes could allow the development of increasingly innovative therapies for both prophylaxis and acute treatment. Significant advances in understanding the pathophysiology of migraine have enabled the development of new drugs in recent years, giving great hope to millions of migraine sufferers. The pathophysiology of migraine is a complex territory still not completely clear. According to electrophysiology and imaging studies, many brain areas are involved. The anatomy and physiology of the nervous structures involved in the genesis of pain and the pathways that regulate them should be analyzed to understand the pathophysiological mechanisms underlying migraine. However, according to the data reported in the CaMEO Study, 41% of patients with chronic migraine were in the care of a physician, and just 25% of these patients got an accurate diagnosis of their migraine [219]. Currently, the prevention of migraine takes advantage of several compounds that exercise different mechanisms of action. Most of them have been discovered to have antimigraine properties by indirect evidence. A primary activity includes inhibiting sodium and calcium channels, attenuating central sensation, suppressing cortical diffusion depression, and modulating GABAergic neurotransmission. On the other hand, CGRP antibodies represent the first treatment developed after systematic research and adapted to the disease.

Migraine is a multifactorial disorder for which several metabolic enhancers have been proven to be efficacious, inducing an improvement of energetic brain processes as reported over the last few years. The main pathophysiological alteration may vary among patients, suggesting the role of genetic factors. Cofactors, such as riboflavin and CoQ10, strengthen mitochondrial OXPHOS processes, improving energetic metabolism in the brain of migraineurs. Some energetic deficits have been reported during the last decades of research. Riboflavin has a favorable efficacy/adverse effect profile and may be indicated in subjects with episodic migraine or in combination with other medications, such as beta-blockers. The ketogenic diet also constitutes a promising strategy. Increasing evidence supports the beneficial effects of a ketogenic diet in migraine prophylaxis. A few studies are available to recommend the prescription of the metabolic compound as first-line treatment options; thus, large randomized clinical trials are needed to support the efficacy of these groups of medications and improve the knowledge about their effects on migraine pathogenesis. It is desirable for broader scientific research on the metabolic face of migraine since new encouraging findings have emerged. Pharmacogenetics is a helpful tool to predict the efficacy of treatments. The complexity that characterizes migraine pathophysiology provides many ideas for research and the opportunity to discover novel strategies for therapy.

Knowing the neurophysiopathological mechanisms and the various neurotransmitters underlying migraine is fundamental in order to be able to identify and personalize therapy both in acute cases and for prophylaxis. Furthermore future studies should be addressed to better evaluate the relationship between inflammation and migraine pathogenesis through a possible parallel comparison with inflammatory pathogenesis of cerebrovascular disease [334,335,336,337,338,339,340,341,342]. 

## Figures and Tables

**Table 1:** Therapeutic options: in this table are indicated drugs, posology and their effects.

Drug	Dose	Potential Mecchanism of Action
**Beta Blocker**		
Propranolol	40 mg to 120 mg b.d.	Inhibition of nitric oxide synthase Interaction with the serotonergic system Inhibition of thalamic relay neurons Block of central sensitization
Metoprolol	25−100 mg twice daily	
**Anticonvulsants**		
Valproate	400−600 mg twice daily	Block of low-threshold T-type calcium ion channels Suppression of proteinkinase C (glumatatergic neurotransmission) Inhibition of the NF-KB pathway Downregulation of CGRP expression
Topiramate	50−200 mg/day	Block of voltage-dependent sodium channels and high-voltage-activated L-type calcium channels Inhibition of glutamate-mediated excitatory neurotransmission Facilitation of GABA-A-mediated inhibition Inhibition of carbonic anhydrase activity Reduction of CGRP secretion from trigeminal neurons
**Calcium Channel Blocker**		
Flunarizine	5−15 mg daily	Block of voltage-gated sodium channels Reduction of neuronal excitability and normalization cortical hyperexcitability D2 antagonism
**Antidepressants**		
Amitriptyline	25−75 mg nocte (start with 10 mg)	Inhibition of serotonin/noradrenaline reuptake pump Facilitation of endogenous pain control mechanisms
Fluoxetine	20 mg daily	Block of serotonin reuptake reversible 5-HT2C receptor blocker
**Onabotulinumtoxin A**	155 units at fixed sites	Block of peripheral and central sensitization Relaxation of head and neck muscles
**Renin Angiotensin System Modulating Compounds**		
Lisinopril	20 g daily	Modulation of vasoreactivity, alteration of sympathetic tone, and promotion of degradation of proinflammatory factors Modulation of endogenous opioid system Reduction of angiotensin-mediated effects Increase of sympathetic discharge Release of adrenal catecholamines Modulation of cerebral RAS influencing neuronal, astrocytic, and endothelial cell activity
Candesartan	16 mg daily	
**Nutraceuticals**		
Riboflavin	400 mg daily	Effect on mitochondria?
Coenzyme Q10	100 mg three times daily or 75 mg twice daily	Effect on mitochondria?
Butterbur	50 to 75 mg twice daily	Anti-inflammatory action through inhibition of cyclooxygenase-2
Feverfew	6.25 mg three times daily	Vasodilatory effects through inhibition of L-type voltage-gated calcium channels Inhibition of Fos-induced activation of trigeminal nucleus caudalis Partial agonist activity at TRPA1 channels Antinociceptive and anti-inflammatory effects
Magnesium	600 mg	Effect on enzymatic function/mitochondria?

**Table 2:** New emerging therapies: summary of trials.

**SPARTAN:** **Three Doses of lasmiditan (50 mg, 100 mg and 200 mg) Compared to Placebo in the Acute Treatment of Migraine**	**Lasmiditan 50 mg/** **Lasmiditan 50 mg**	**Lasmiditan 50 mg/** **Placebo**	**Lasmiditan 100 mg/** **Lasmiditan 100 mg**	**Lasmitidan 100 mg/Placebo**	**Lasmiditan 200 mg** **/Lasmiditan 200 mg**	**Lasmitidan 200 mg/Placebo**	**Placebo/Placebo**
	pain freedom at 2 h with 50 mg: 28.6%		pain freedom at 2 h with 100 mg: 31.4%		pain freedom at 2 h with lasmiditan 200 mg: 38.8%		placebo 21.3%
**SAMURAI:** **Lasmiditan Compared to Placebo in the Acute Treatment of Migraine**	**Lasmiditan 100 mg**		**Lasmiditan 200 mg**			**Placebo**	
	100 mg lasmiditan administered PO within 4 h of onset of migraine attack. If the migraine did not respond 2 h after the dose, a second dose of study drug can be taken up to 24 h after the first dose Pain freedom at 2 h: 28.2%		200 mg lasmiditan administered PO within 4 h of onset of migraine attack. If the migraine did not respond 2 h after the dose, a second dose of study drug can be taken up to 24 h after the first dose Pain freedom at 2 h: 32.2%			Placebo administered PO within 4 h of onset of migraine attack. If the migraine did not respond 2 h after the dose, a second dose of study drug can be taken up to 24 h after the first dose Pain freedom at 2 h: 15.3%	
**ACHIEVE I:** **Efficacy, Safety, and Tolerability Study of Oral Ubrogepant in the Acute Treatment of Migraine**	**Placebo**		**Ubrogepant 50 mg**			**Ubrogepant 100 mg**	
	2 placebo-matching ubrogepant 50 mg tablets, orally for treatment of a qualifying migraine attack Pain freedom at 2 h was reported by 11.8%		1 ubrogepant 50 mg tablet and 1 placebo-matching ubrogepant 50 mg tablet, orally for treatment of a qualifying migraine attack Pain freedom at 2 h was reported by 19.2%			2 Ubrogepant 50 mg tablets, orally for treatment of a qualifying migraine attack Pain freedom at 2 h was reported by 21.2%	
**ACHIEVE II:** **Efficacy, Safety, and Tolerability of Oral Ubrogepant in the Acute Treatment of Migraine**	**Placebo**		**Ubrogepant 25 mg**			**Ubrogepant 50 mg**	
	1 placebo-matching ubrogepant tablet, orally for treatment of a qualifying migraine attack Pain freedom at 2 h was reported by 65 of 456 (14.3%)		1 ubrogepant 25 milligram (mg) tablet, orally for treatment of a qualifying migraine attack Pain freedom at 2 h was reported by 90 of 435 (20.7%)			1 ubrogepant 50 mg tablet, orally for treatment of a qualifying migraine attack. Pain freedom at 2 h was reported by 101 of 464 participants (21.8%)	
**GLADIATOR:** **An Open-label, Long-term, Safety Study of Lasmiditan for the Acute Treatment of Migraine**	**Lasmiditan 100 mg**			**Lasmiditan 200 mg**			
	Participants received oral dose of 100 milligrams (mg) lasmiditan within 4 h of onset of migraine attack. If the migraine did not respond within 2 h after first dose or if responded and recurred then a second dose was permitted within 24 h after first dose Pain freedom was observed in 26.9%			Participants received oral dose of 200 mg lasmiditan within 4 h of onset of migraine attack. If the migraine did not respond within 2 h after first dose or if responded and recurred then a second dose was permitted within 24 h after first dose Pain freedom was observed in 32.4%			

## Abbreviations

CRP	C-reactive protein
IL	interleukin
TNF	tumor necrosis factor
CGRP	calcitonin gene-related peptide
MO	migraine without aura
SP	substance P
MZ	monozygotic twins
DZ	dizygotic twins
HIS	International Headache Society
GWAS	genome-wide association studies
FHM	familial hemiplegics
ATP	adenosine triphosphate
CACNA1A	calcium voltage-gated channel subunit alpha1 A
GWA	genome-wide association meta-analysis
SNP	single nucleotide polymorphism loci
WGHS	Women’s Genome Health Study
FGF6	fibroblast growth factor 6
HPSE2	heparanase 2
NPFF	neuropeptide precursor of the FF-amide peptide
ASIC	acid-sensitive ion channels
CSD	cortical diffusion depression
NSAIDs	nonsteroidal anti-inflammatory drugs
DHE	dihydroergotamine
5-HT	5-hydroxytryptamine
NF-κB	nuclear factor kappa-light-chain-enhancer
SNAP	synaptosomal-associated protein
ACE	angiotensin-converting enzyme inhibitors
ARB	angiotensin receptor blocker
MTHFR	methylenetetrahydrofolate reductase
MTRR	methionine synthase reductase
CoQ10	coenzyme Q10
NMDA	N-methyl-D-aspartate
MMD	migraine days monthly
CGRP	calcitonin gene-related peptide
CNS	central nervous system
PNS	peripheral nervous systems
LC	locus coeruleus
PAG	periaqueductal grey matter
VIP	vasoactive intestinal polypeptide
VPAC1 and VPAC2	vasoactive intestinal polypeptide receptors 1 and 2
PACAP	pituitary adenylate cyclase-activating polypeptide
SPG	parasympathetic sphenopalatine ganglia
MCAs	middle cerebral arteries
MMAs	middle meningeal arteries
NPY	neuropeptide Y
TRIG	trigeminal ganglia
